# Gastrointestinal parasites of *Peltocephalus dumerilianus* (Testudines: Podocnemididae) from Jaú National Park, Brazilian Amazon

**DOI:** 10.1590/S1984-29612024013

**Published:** 2024-02-12

**Authors:** Luciana Raffi Menegaldo, Carmen Andrea Arias-Pacheco, Patricia Parreira Perin, José Hairton Tebaldi, Estevam Guilherme Lux Hoppe

**Affiliations:** 1 Laboratório de Enfermidades Parasitárias - LabEPar, Departamento de Patologia, Reprodução e Saúde Única - DPRSU, Faculdade de Ciências Agrárias e Veterinárias - FCAV, Universidade Estadual Paulista - UNESP, Jaboticabal, SP, Brasil

**Keywords:** Big-headed Amazon River turtle, helminths, morphology, neotropics, parasitology, Cabeçudo, helmintos, morfologia, neotrópicos, parasitologia

## Abstract

The big-headed Amazon River turtle, *Peltocephalus dumerilianus*, is endemic to the Orinoco and Amazon River basins. It is a food source for local communities, often unsustainably. Knowledge about *P. dumerilianus’* parasitological fauna and host-parasite relationships is limited. Thus, ecological aspects of gastrointestinal parasitism in this species were investigated. Helminths were found in the gastrointestinal tract of 21 turtles, morphologically identified, and infection descriptors calculated. All animals harbored helminths: nematodes *Ancyracanthus pinnatifidus*, *Paratractis hystrix*, *Atractis trematophila*, *Klossinemella conciliatus* indeterminate three *Klossinemella* species, and digeneans *Nematophila grandis*, *Helicotrema spirale*, and *Telorchis hagmanni*. The highest parasite load occurred in the large intestine, followed by the small intestine and stomach. Shell length directly correlated with parasite burden of heteroxenic helminths, with males having higher burden than females. This is the first record of *A. trematophila*, *K. conciliatus*, and *T. hagmanni* in *P. dumerilianus*, and new location record for *A. trematophila*, *P. hystrix*, *N. grandis*, *H. spirale*, and *T. hagmanni*. Three potentially new *Klossinemella* species are presented.

## Resumo

O cabeçudo, *Peltocephalus dumerilianus*, é endêmico das bacias dos rios Orinoco e Amazonas. Ele é uma fonte de alimento para as comunidades locais, frequentemente de maneira insustentável. O conhecimento sobre a fauna parasitológica de *P. dumerilianus* e suas relações hospedeiro-parasito são limitados. Assim, investigou-se os aspectos ecológicos do parasitismo gastrointestinal nessa espécie. Helmintos encontrados no trato gastrointestinal de 21 tartarugas, foram identificados morfologicamente e descritores de infecção calculados. Todos os animais abrigavam helmintos: nematoides Ancyracanthus *pinnatifidus*, *Paratractis hystrix*, *Atractis trematophila*, *Klossinemella conciliatus* e três espécies de *Klossinemella* indeterminados, além dos digenéticos *Nematophila grandis*, *Helicotrema spirale* e *Telorchis hagmanni*. A maior carga parasitária ocorreu no intestino grosso, seguida pelo intestino delgado e estômago. O comprimento da carapaça correlacionou-se diretamente com a carga de parasitos de helmintos heteroxênicos, e os machos apresentaram uma carga parasitária maior que as fêmeas. Este é o primeiro registro de *A. trematophila*, *K. conciliatus* e *T. hagmanni* em *P. dumerilianus*, e um novo registro de local para *A. trematophila*, *P. hystrix*, *N. grandis*, *H. spirale* e *T. hagmanni*. Três potenciais novas espécies de *Klossinemella* são apresentadas.

## Introduction

The big-headed Amazon River turtle, *Peltocephalus dumerilianus* (Schweigger, 1812) (Testudines: Podocnemididae), is endemic to the Orinoco and Amazon River basins, primarily inhabiting blackwater rivers. It serves as a food source for local traditional communities, often subjected to unsustainable harvesting practices (Parra-Henao et al., 2019; Gentil et al., 2021). This turtle species has been categorized as “Vulnerable” in the IUCN Red List of threatened species (2016) and is listed in Appendix II of The Convention on International Trade in Endangered Species of Wild Fauna and Flora (Thomson, 2021). In contrast, the Chico Mendes Institute of Biodiversity Conservation from Brazil designates this species as of “Least Concern” (ICMBio, 2018).

However, knowledge about the parasitological fauna and host-parasite relationships within Podocnemididae remains limited. While a few studies have reported the presence of nematodes such as *Paratractis hystrix* (Sarmiento, 1959) and *Ancyracanthus pinnatifidus* (Guerrero, 2021), and digeneans *Nematophila grandis* and *Helicotrema spirale* (Fernandes & Kohn, 2014) in *P. dumerilianus*; none have explored the associations between host characteristics and its parasites. The study of parasites has often been used to successfully evaluate conservation problems, because they may reveal information about hosts and their environment (Gagne et al., 2022). Anthropogenic alterations such as overexploitation or habitat degradation could diminish population genetic diversity, and genetically homogeneous hosts are recognized to be more susceptible to infection (Ekroth et al., 2019). Vulnerable species are usually more prone to diseases, which makes it essential to include disease prevention and risk management to the formulation of management and conservation strategies (Bower et al., 2019). Therefore, this study aims to describe species that parasitize the gastrointestinal system of the big-headed Amazon River turtle, providing information that could be the starting point for a better understanding of the parasite-host relationships for this species.

## Material and Methods

### Study area and sampling

Big-headed Amazon River turtles are still relatively abundant in the Rio Negro River basin and because these reptiles are easy to capture, they are unsustainable hunted throughout the year to obtain mainly meat and eggs.

**Figure 1:**
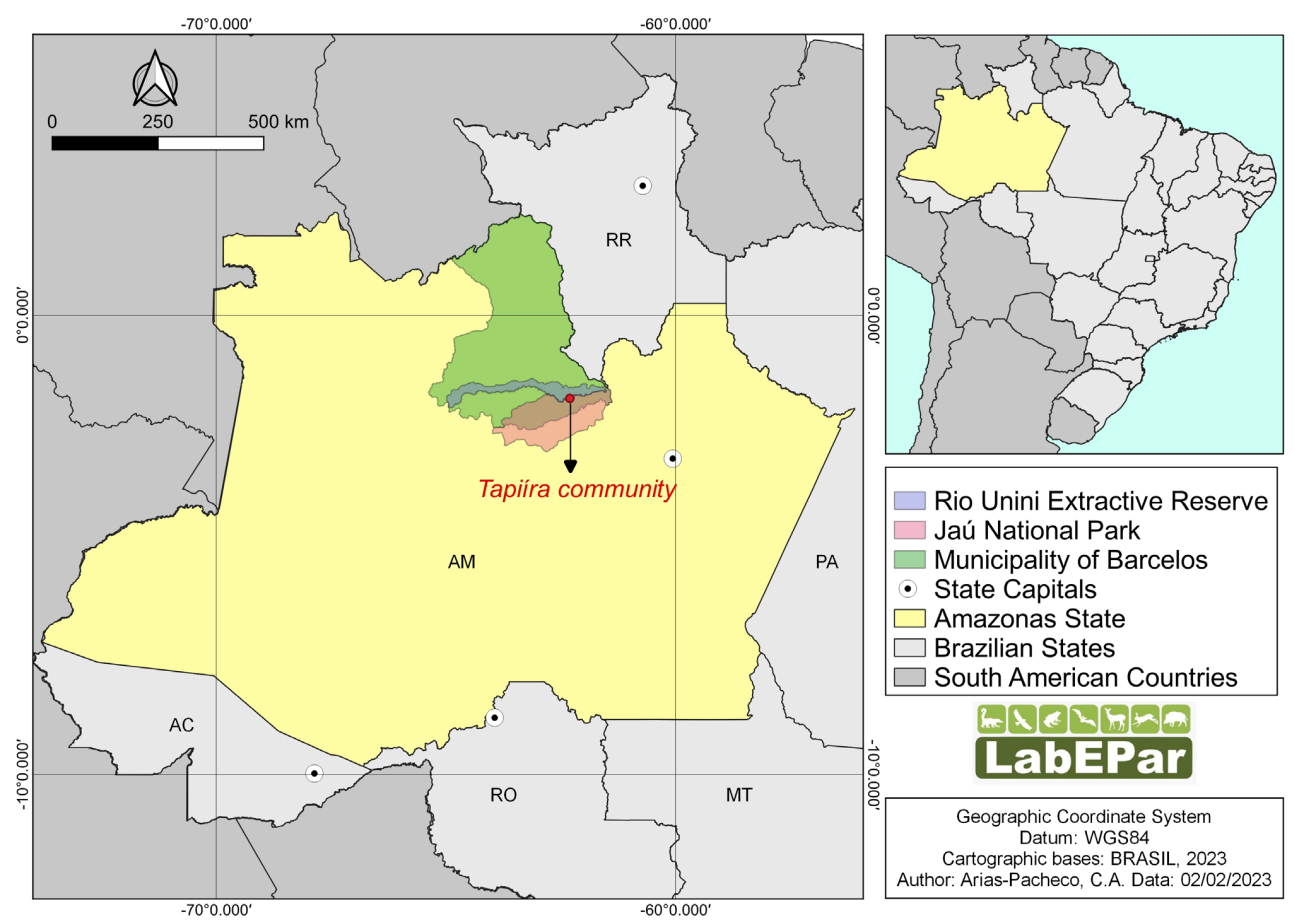
Geographical location of the Tapiíra community in Jaú National Park, Barcelos municipality, State of Amazonas, Brazil.

The Jaú National Park is a Brazilian conservation unit for nature protection, belonging to the Lower Negro River Mosaic. The Tapiíra (Figure 1) is a riverside community on the Unini River in Barcelos municipality, Amazonas state (1º45'49”S and 62º13'29”W), located within Jaú National Park. The economies of their human populations are based on native plants, fishing, hunting, and agricultural activities (FVA, 2011). The Amazon is the largest and most well-preserved biome in Brazil. It accounts for a significant proportion of the world’s biodiversity and has the largest hydrographic basin on our planet. Most of the Amazon biome is located in the state of Amazonas (Módolo et al., 2019).

The viscera from 21 big-headed Amazon River turtles’ subsistence-hunted by community inhabitants were collected. Immediately after evisceration, the gastrointestinal tract was tied and anatomically separated into stomach, small intestine, and large intestine. Each portion was slit-open, and the total content was washed through metal sieves. The residues left on the sieves were stored in plastic bottles and fixed in Railliet and Henry solution (5% formaldehyde, 3% acetic acid, and 0.9% saline q.s.p.). The samples were transported at room temperature to the Parasitic Diseases Laboratory (LabEPar), FCAV/Unesp, Jaboticabal, São Paulo, for further analyses.

### Parasitological assessment

In the laboratory, all gastrointestinal contents were screened under a stereoscopic microscope (Leica EZ4 HD; Leica Microsystems Limited, Wetzlar, Germany) to identify the parasites. For taxonomic identification, the collected helminths were clarified in 80% acetic acid and diaphanized in beechwood creosote, if necessary. Subsequently, they were mounted on temporary glass slides and observed under an Olympus BX-51 microscope equipped with a Q-Color3 camera. Photomicrographs were processed using Image-Pro Plus 6.0 image analyzer software. Species were identified according to the taxonomic keys published by Diesing (1839, 1850, 1851), Travassos (1934), Costa (1961), and Fernandes & Kohn (2014). All measurements have been expressed in millimeters as mean (mm) ± standard deviation, unless otherwise stated, and were based on mature specimens. Illustrations of each helminth species were obtained using a Carl Zeiss microscope equipped with a camera lucida. Voucher specimens were deposited in the Helminthological Collection of the Oswaldo Cruz Institute (CHIOC - Fiocruz, Rio de Janeiro). The remaining samples were stored in the Helminthological Collection of LabEPar, FCAV/Unesp (Jaboticabal, São Paulo, Brazil).

### Statistical analysis

The descriptors of infection (prevalence, mean abundance, mean intensity, and intensity range) were calculated as described by Bush et al. (1997). The Kolmogorov-Smirnov test was used to evaluate the goodness-of-fit for all data. The Pearson’s chi-square test was used to compare the effects of parasite intensity on host weight and shell length. The Mann-Whitney U test was used to compare the parasite intensity between sexes (males vs. females). All statistical analyses were performed using the GraphPad Prism 5.0 software, with P-value set to 0.05.

## Results and Discussion

**Figure 2:**
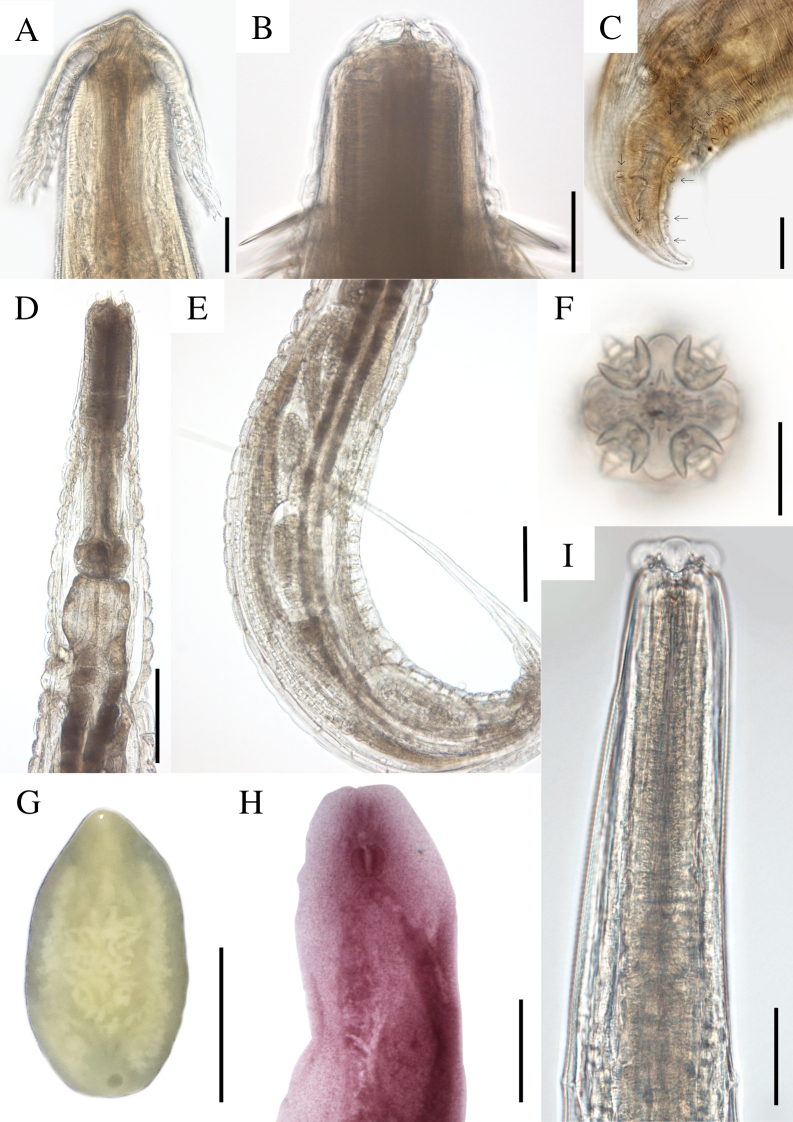
Gastrointestinal parasites from *Peltocephalus dumerilianus* from the Jaú National Park, Barcelos, State of Amazonas. A. *Ancyracanthus pinnatifidus*, anterior end. B. *Paratractis hystrix* anterior end. C. *Paratractis hystrix*, posterior end of the male with two pairs of ventral pre-cloacal papillae, three pairs of ad-cloacal papillae, and five pairs of post-cloacal papillae. D. *Atractis trematophila*, anterior end. E. *Atractis trematophila*, posterior end of the female. F. *Klossinemella conciliatus*, apical view of the anterior end. G. *Nematophila grandis*, whole view. H. *Helicotrema spirale*, anterior end. Scale: A, B, D, E - 50 µm; C - 60 µm; F - 20 µm; G - 10mm; H - 500 µm; I - 50 µm.

All the analyzed animals were found to be parasitized by helminths; ten species, representing seven genera, five families, and five orders, were identified (Figure 2). We identified seven Nematoda species, including one species from the Gnathostomatidae family, namely *Ancyracanthus pinnatifidus* (Diesing, 1839), and six species from the Atractidae family, namely *Paratractis hystrix* (Diesing, 1851), *Atractis trematophila* (Travassos, 1934), *Klossinemella conciliatus* (Alho, 1964), and three indeterminate *Klossinemella* species. Three Digenea species belonging to the Cladorchiidae, Liolopiidae, and Telorchiidae families, namely *Nematophila grandis* (Diesing, 1839), *Helicotrema spirale* (Diesing, 1850), and *Telorchis hagmanni* (Lent & Freitas, 1937), respectively, were also identified.

The highest parasite load was observed in the large intestine, followed by the small intestine and stomach. All nematodes, except *A. pinnatifidus*, were observed in all anatomical segments of the digestive tract, but with greater counts in the large intestine. Superficial macroscopic lesions were observed only in the gastrointestinal mucosa where Digenea *N. grandis* was attached. Data related to parasitic infections are summarized in Table 1.

**Table 1:** Prevalence (%),Confidence Interval (CI), mean abundance, mean intensity, and range of intensity of gastrointestinal parasites in *Peltocephalus dumerilianus* from the Jaú National Park, Barcelos, State of Amazonas, Brazil.

Parasite	Recovered from	Prevalence (%)	Abundance	Mean Intensity	Range of Intensity
Nematoda					
Atractidae					
*Paratractis hystrix*	S, SI, LI	9.52%	51.61	542	18 - 1,066
(CHIOC 3813)		(CI: 2.65 - 28.91%)			
*Atractis trematophila*	S, SI, LI	95.24%	123,566.50	129,744.50	8,125 - 467,800
(CHIOC 38129)		(CI: 77.33 - 99.15%)			
*Klossinemella conciliatus*	S, SI, LI	100%	2,806,281.05	2,806,281.05	78,666 - 6,690,317
(CHIOC 38131)		(CI: 84.54 - 100%)			
*Klossinemella* sp. 01	S, SI, LI	100%	104,161.76	104,161.76	2,260 - 270,538
(CHIOC 38132)		(CI: 84.54 - 100%)			
*Klossinemella* sp. 02	S, SI, LI	80.95%	55,411.29	68,449.24	2,000 - 290,000
(CHIOC 38133)		(CI: 59.99 - 92.33%)			
*Klossinemella* sp.03	S, SI, LI	100%	1,499,208.52	1,499,208.52	54,000 - 3,052,600
(CHIOC 38134)		(CI: 84.54 - 100%)			
Gnathostomatidae					
*Ancyracanthus pinnatifidus* (CHIOC 38128)	S	57.14%	1	1.75	01/mar
		(CI: 36.55 - 75.53%)			
Digenea					
Cladorchiidae					
*Nematophila grandis*	S, SI, LI	100%	45.05	45.05	1 - 351
(CHIOC 38135)		(CI: 84.54 - 100%)			
Liolopidae					
*Helicotrema spirale*	SI	66.67%	33.19	49.79	2 - 209
(CHIOC 38130)		(CI: 45.37 - 82.80%)			
Telorchiidae					
*Telorchis hagmanni*	SI	4.76%	0.14	3	-
(CHIOC 38137)		(CI: 0.84 - 22.67%)			

S: stomach; SI: small intestine; LI; large intestine.

### *Ancyracanthus pinnatifidus* Diesing, 1839 (Gnathostomatidae: Ancyracanthinae)

Cylindrical body with transverse-grooved cuticles. Anterior end with two pairs of identical chitinized appendages that were branched, feather-like, and directed backwards (Figure 2). Mouth with two lips. The esophagus was muscular in its first and glandular in its second portion. The nerve ring was observed at the beginning of the esophagus. Males with two equal spicules. Three pairs of pre- and one pair of post-cloacal papillae. Females larger than males. Didelphic, prodelphic, and oviparous females. Vulva in the posterior third of the body. The eggs were elliptical, small, and had external fine granulations. Morphometric data are not available because only two mature individuals of each sex were found. *Ancyracanthus pinnatifidus* was the only species of the Gnathostomatidae family and was the most prevalent parasite species found in the stomach (57.14%, CI: 36.55-75.53%). It was originally described as parasitizing the digestive system of *Podocnemis expansa* (Schweigger, 1812) and *Podocnemis unifilis* (Troschel, 1848) in State of Amazonas, Brazil (Diesing, 1839) and was also reported in *P. dumerilianus* from the same state (Guerrero, 2021). In contrast to the original description that characterized the helminth as having a glandular esophagus throughout its entire length, we observed a small muscular portion in the first part of its esophagus (Figure 2).

### *Paratractis hystrix* (Diesing, 1851) (Cosmocercoidea: Atractidae)

**Figure 3:**
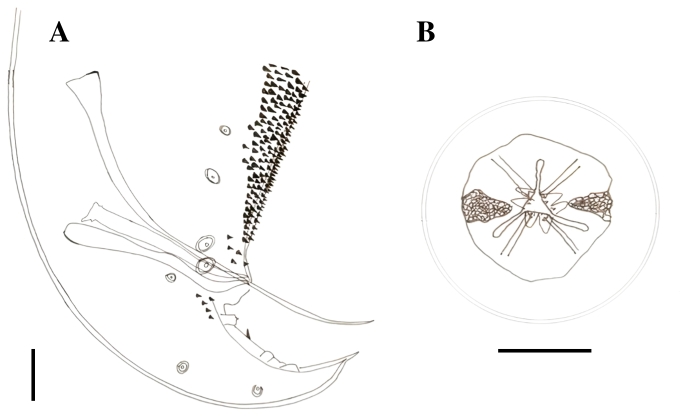
*Paratractis hystrix*, State of Amazonas, Brazil. A. Posterior extremity of the male with two pairs of ventral precloacal papillae; three ad-cloacal pairs, two ventral and one ventrolateral; and five more post-cloacal pairs, three ventral and two lateral pairs. B. Mouth with three lips, one dorsal with two papillae and two sub-ventral, each containing one papilla and one amphid. Scale: A and B: 0.05 mm.

Elongated cylindrical body, mostly covered by conical spines with thinner ends, except in the anterior region close to the mouth and dorsal region in adult males. Spine arrangement differs between males and females. The mouth had three lips, one dorsal with two papillae, and two subventral lips, each containing a papilla and an amphid. Each papilla had small projections close to the inner margin of the lip. The esophagus was observed to be divided into two parts: the anterior part, which was cylindrical and muscular, with a strongly chitinized lumen, and the posterior part, which was glandular, ending in a bulb with a valve apparatus. A post-esophageal excretory pore was observed. Males with slightly curved anterior ends, uneven spicules, and with gubernacule. Females with their vulva close to the anus. Tail constricted behind the anus, forming a short conical process, especially in the females. Viviparous females with larvae at an advanced developmental stage (Figure 2, Figure 3).

Males (n=10): Total length 7.180 mm ± 0.544, body width at junction of esophagus to intestine 0.287 mm ± 0.014, anterior esophagus length 0.391 mm ± 0.012, posterior esophagus length 0.364 mm ± 0.144. The distance from the excretory pore and cervical papillae to the anterior end is 1.149 mm ± 0.116 and 0.285 mm ± 0.011, respectively. The spicules are visible dissimilar in size, being 0.167 mm ± 0.019 and 0.375 mm ± 0.028 long. Gubernacule is 0.181 mm ± 0.018 long. At the posterior end are two pairs of ventral pre-cloacal papillae, three ad-cloacal pairs, being two ventral and one ventrolateral, and five post-cloacal pairs, being three ventral pairs and two lateral pairs.

Females (n=10): Total length 6.430 mm ± 0.501, body width at junction of esophagus to intestine 0.346 mm ± 0.013, length of anterior and posterior portions of esophagus are 0.390 mm ± 0.024 and 0.361 mm ± 0.016, respectively. The distance from the excretory pore and cervical papillae to the anterior end is 1.149 mm ± 0.116 and 0.271 mm ± 0.026, respectively. The distance from vulva to anus is 0.089 mm ± 0.018 and from vulva to posterior end is 0.325 mm ± 0.038.

*Paratractis hystrix* was first described parasitizing the large intestine of the red-headed Amazon side-necked turtle, *Podocnemis erytrocephala* (Spix, 1824) as *Ascaris hystrix* (Diesing, 1851) based on the presence of three lips characteristic of the genus *Ascaris.* It was redescribed as *Atractis hystrix* (Von Drasche, 1882), because of its esophageal division, unequal spicules, and the number of caudal papillae. Finally, the *Paratractis* genus was stablished (Sarmiento, 1959), which differs from the genus *Atractis* in terms of the number of lips. *P. hystrix* has also been described in the large intestine of the big-headed Amazon River turtles from Pucallpa, Peru (Sarmiento, 1959). This is a new location record for this species.

### *Atractis trematophila* Travassos, 1934 (Cosmocercoidea: Atractidae)

**Figure 4:**
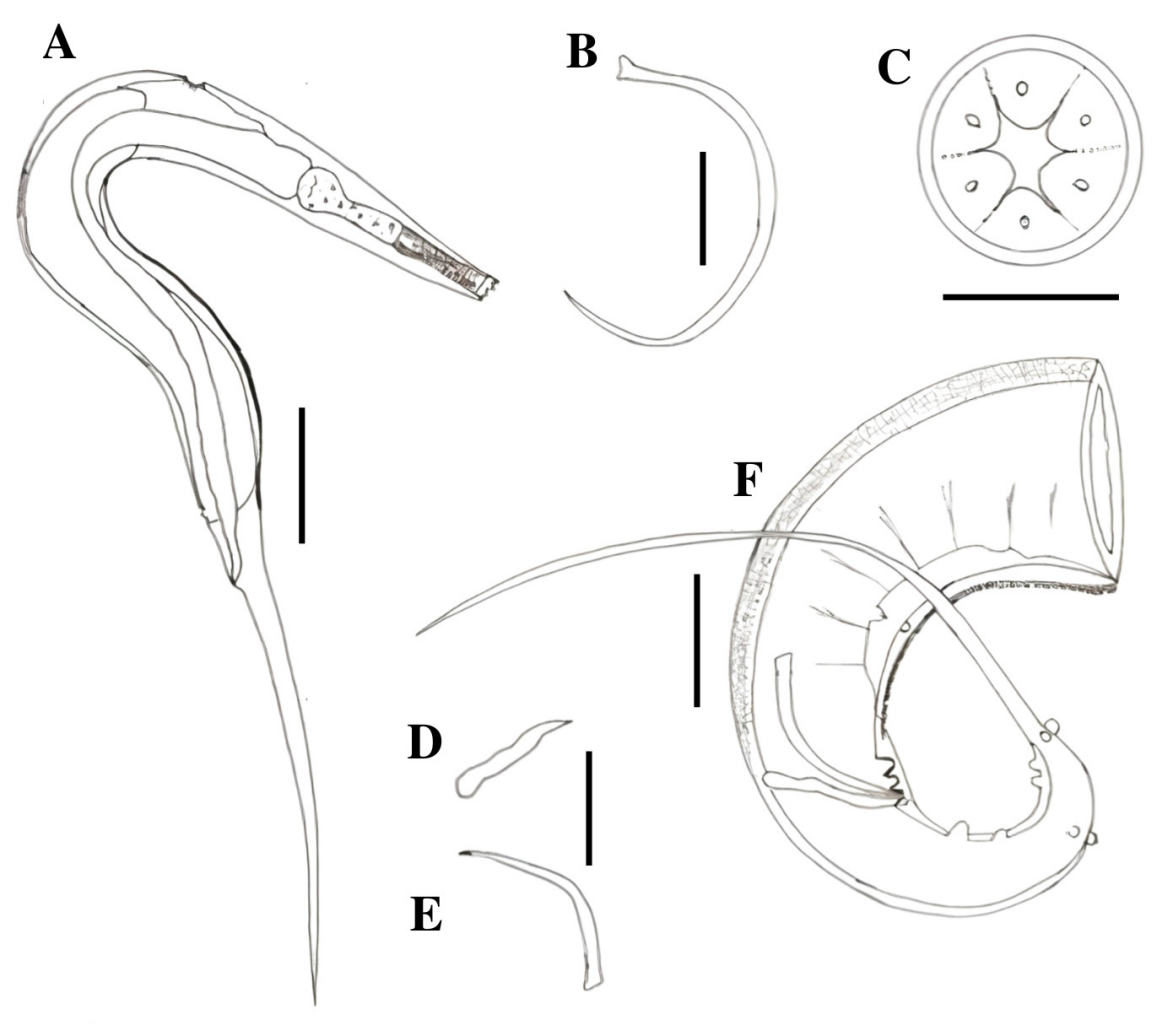
*Atractis trematophila*, Amazonas State, Brazil. A. apical view of the mouth with six indistinct lips. B. Female specimen, detail of the esophagus characteristic of the genus. C. Large spicule. D. Gubernacule. E. Small spicule. F. Posterior extremity of the male with one pair of ventral pre-cloacal papillae, three pairs of ventral ad-cloacal and six more pairs of post-cloacal papillae. The posterior ventral region of the body has small spines aligned in three or four rows ending near the cloacal region. Scale: A, C, D, E, F: 0.05 mm; B: 0.2 mm.

Fusiform cylindrical body with finely grooved cuticle and mouth with six lips. The esophagus was cylindrical and muscular, with a strongly chitinized lumen in its anterior portion, surrounded by a nerve ring, and ended in a bulb equipped with a valve apparatus. Presence of a post-esophageal excretory pore. Males with spirally curved posterior ends. Tails constricted behind the anus, forming a long subulate conical process. Uneven spicules. Gubernacule present. Females with elongated, conical, or subulate tails. The vulva was located close to the anus and the females were viviparous (Figure 4).

Males (n=10): Total length 1.827 mm ± 0.076, body width at junction of esophagus to intestine 0.068 mm ± 0.003, anterior esophagus length 0.162 mm ± 0.005, posterior esophagus length 0.156 mm ± 0.007. The distance from the excretory pore to the anterior end is 0.501 mm ± 0.055. The spicules are visible dissimilar in size, the smaller one is 0.078 mm ± 0.007 long and less chitinized than the larger, that is 0.220 mm ± 0.014 long and striated. Gubernacule is 0.052 mm ± 0.005 long. The distance from the anus to the posterior end is 0.375 mm ± 0.027. Posterior end with three pairs of ventral pre-cloacal papillae, one ventral ad-cloacal pair and six pairs of post-cloacal papillae, being four ventral pairs and two lateral pairs. Posterior ventral region of the body has small spines aligned in three or four rows close to the cloacal region.

Females (n=10): Total length 2.019 mm ± 0.156, body width at junction of esophagus to intestine 0.084 mm ± 0.011, length of anterior and posterior portions of esophagus are 0.167 mm ± 0.017 and 0.164 mm ± 0.012, respectively. The distance from the excretory pore to the anterior end is 0.532 mm ± 0.069. The distance from vulva to anus is 0.099 mm ± 0.008 and from vulva and anus to posterior end is 0.696 mm ± 0.066 and 0.537 mm ± 0.217, respectively.

*Atractis trematophila* was first described as hyperparasitizing the digenean *Nematophila grandis* in another Amazon River turtle as well as parasitizing the digestive system of the same turtle (Travassos, 1934). This is the first report of *A. trematophila* in the stomach, large and small intestine of *P. dumerilianus*, and a new location record for this species. The presence of six lips, each with a papilla, and the viviparity of this parasite are the two differences observed between the description given by Travassos (1934) and our description. Dujardin (1845) also observed the characteristic six lips. Similar to the report by Bursey & Flanagan (2002), we corroborated the viviparity of this parasite. We observed that larvae appeared curved and tangled in the female uterus, providing a false impression of the presence of eggs.

### *Klossinemella conciliatus* Alho, 1964 (Cosmocercoidea: Atractidae)

**Figure 5:**
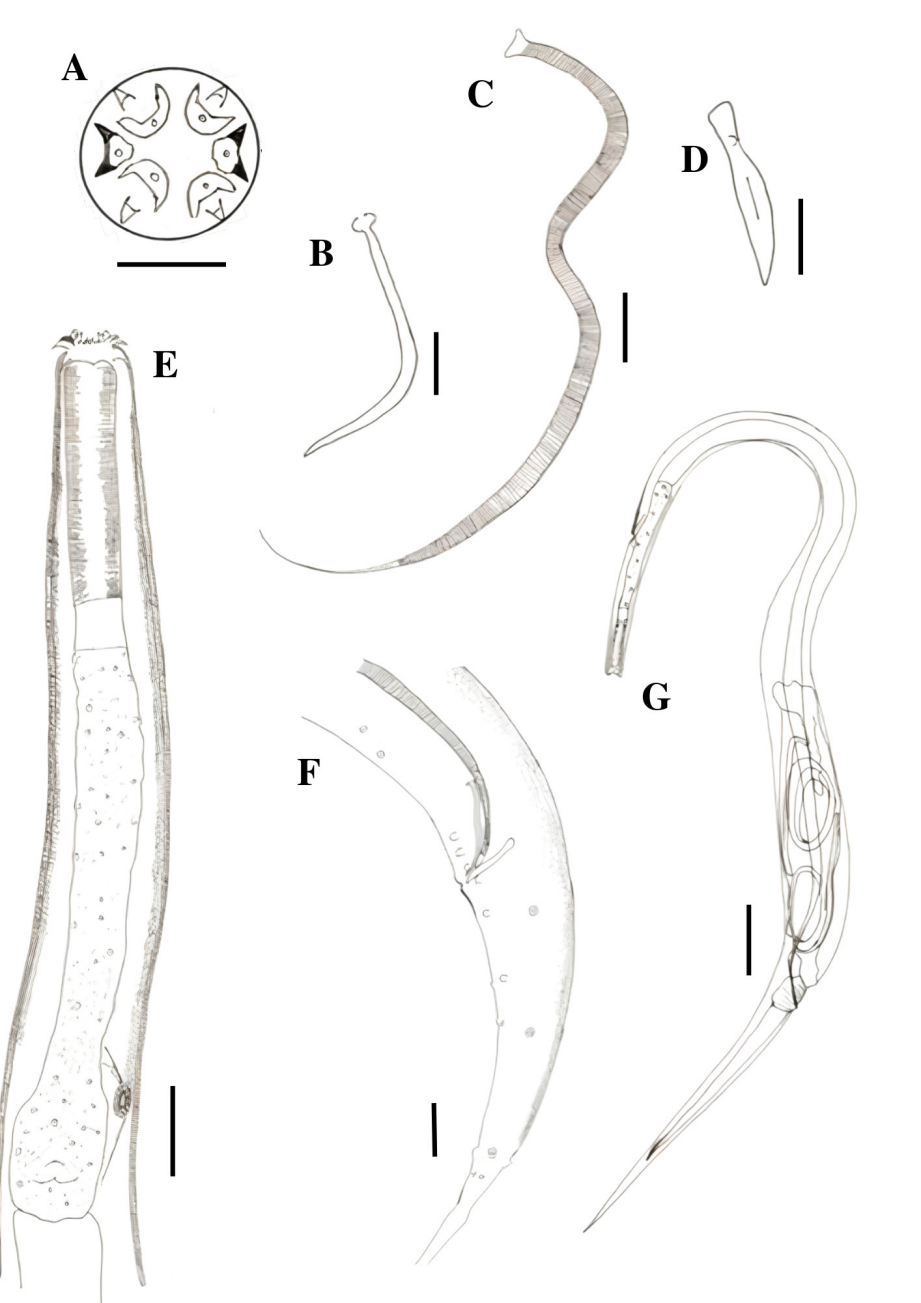
*Klossinemella conciliatus*, State of Amazonas, Brazil. A. Apical view of mouth with Y-shaped chitinized structures. B. Minor spicule. C. Striated major spicule. D. gubernacle. E. Anterior end of the esophagus with a detail of the bulbar region and excretory pore in the region of the bulb. F. Posterior extremity of the posterior end of male with presence of two pairs of ventral precloacal papillae, four ventral ad cloacal pairs, five ventral post cloacal pairs and three ventrolateral post cloacal pairs. G. Prodelphic female specimen with larvae inside. Scale: A, B, D: 0.025 mm; C, E, F: 0.05 mm; G: 0.2 mm.

Mouth with two bilobed lips, each lobe being a small recess forming smaller lobes. Chitinized Y-shaped structures interpreted as highly modified interlips. Discrete division of the muscular and glandular esophagus and poorly developed bulb. Nerve ring located at the end of the anterior esophagus. Presence of lateral alae. Excretory pore located in the final third of the posterior esophagus. Males with two unequal spicules. Gubernacule present. Females with vulva close to the anus and viviparous (Figure 5).

Males (n=10): Total length 3.424 mm ± 0.329, body width at junction of esophagus to intestine 0.079 mm ± 0.009, anterior esophagus length 0.178 mm ± 0.018, posterior esophagus length 0.383 mm ± 0.038. The distance from the excretory pore and cervical papillae to the anterior end is 0.424 mm ± 0.052 and 0.082 mm ± 0.009, respectively. The small spicule is 0.129 mm ± 0.015 long and the large spicule is more chitinized and striated, 0.576 mm ± 0.073 long. Gubernacule is 0.067 mm ± 0.005 long. The distance from the anus to the posterior end is 0.684 mm ± 0.103. Posterior end with five pairs of ventral pre-cloacal papillae, one ventral ad-cloacal pair and eight pairs of post-cloacal papillae, being five ventral and three laterals.

Females (n=10): Total length 3.649 mm ± 0.231, body width at junction of esophagus to intestine 0.089 mm ± 0.011, length of anterior and posterior portions of esophagus are 0.149 mm ± 0.030 and 0.399 mm ± 0.032, respectively. The distance from the excretory pore and cervical papillae to the anterior end is 0.444 mm ± 0.059 and 0.083 mm ± 0.013, respectively. The distance from vulva to anus is 0.072 mm ± 0.016. Length from vulva and anus to posterior end is 0.931 mm ± 0.059 and 0.859 mm ± 0.048, respectively.

The genus *Klossinemella* comprises the intestinal parasites of freshwater fish and turtles; however, a systematic analysis of this genus has not been undertaken yet. *Klossinemella conciliatus* has been found in the stomach of giant South American turtles (*P. expansa*) in the Amazonas state (Costa et al., 1968). The species from turtles and fishes can only be distinguished based on the size of spicules, gubernacule, and the number of caudal papillae (Costa, 1960, 1961; Costa et al., 1968). *Klossinemella conciliatus* has also been reported in the stomachs of yellow-spotted Amazon River turtles (*Podocnemis unifilis*) from Loreto and Ucayali in Peru (Salízar & Sanchez, 2004). The mouth structures and caudal papillae are the main features of this genus (Moravec & Thatcher, 1997; Costa, 1961). There is some controversy regarding the esophageal division of parasites belonging to this genus. Costa (1961) divided the esophagus into two well-defined portions, including a bulbous dilation provided with valves, whereas Moravec & Thatcher (1997), when redescribing *Klossinemella iheringi*, mentioned the bulb region as indistinct. In our study, although we observed an esophageal division, the bulbar region was slightly wider than the posterior esophagus. This is the first record of *K. conciliatus* in *P. dumerilianus*.

### *Klossinemella* sp. 1 (Cosmocercoidea: Atractidae)

**Figure 6:**
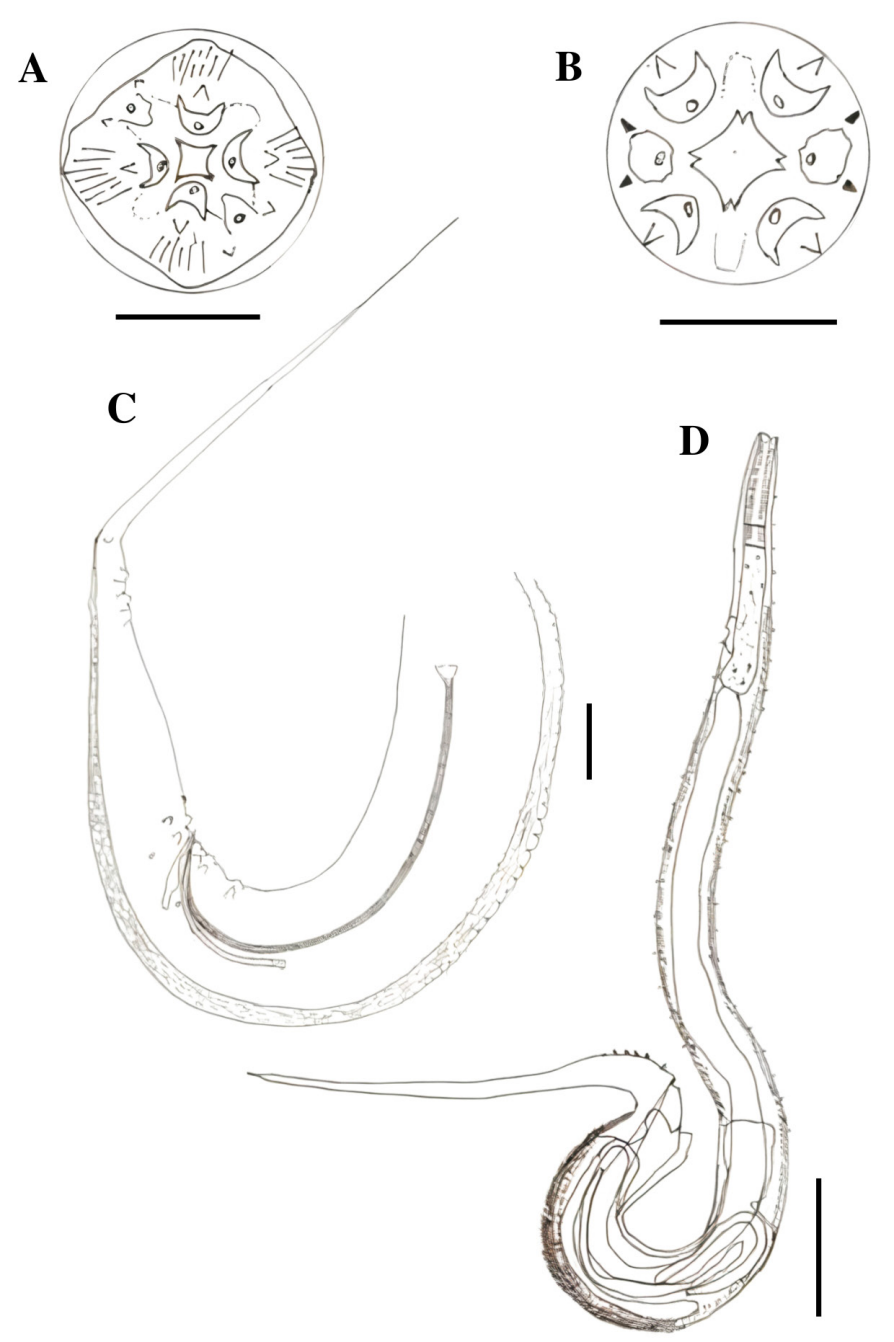
*Klossinemella* sp. 1 and *Klossinemella* sp. 2. A. *Klossinemella* sp. 01, apical view of mouth with Y-shaped chitinized structures. B. *Klossinemella* sp. 02, apical view of mouth with chitinized Y-shaped structures. C. *Klossinemella* sp. 01, posterior end of male with presence of a pair of ventral pre-cloacal papillae, four pairs of ventral ad-cloacal and two pairs of lateral ad-cloacal papillae, and five terminal papillae arranged in two pairs and one near the insertion of the tail. D. *Klossinemella* sp. 2, female prodelphic specimen with larvae inside, detail of the presence of spines in the posterior dorsal region and esophagus with a discrete bulbar region and excretory pore in the bulb region. Scale: A, B: 0.025 mm; C: 0.05 mm; D: 0.2 mm.

Cylindrical body with a transversely striated cuticle and large lateral wings that begin at the level of the posterior esophagus and continue along the body until the insertion of the tail. Mouth with two bilobed lips, each lobe with a small indentation in the form of smaller lobes. A Y-shaped formation, similar to highly modified interlips, observed between the lips. Nerve ring located in the initial portion of the posterior esophagus. Cervical papillae in the second portion of the esophagus. Excretory pore in the final third of the posterior esophagus. Males with two very unequal spicules. Gubernacule present. Females with vulva close to the anus and viviparous. Males with their posterior ends curled. Males and females with a triangular, elongated, and filament-like tail (Figure 6).

Males (n=10): Total length 3.610 mm ± 0.267, body width at junction of esophagus to intestine 0.383 mm ± 0.121, anterior esophagus length 0.190 mm ± 0.010, posterior esophagus length 0.350 ± 0.057. The distance from the excretory pore and cervical papillae to the anterior end is 0.460 mm ± 0.049 and 0.160 mm ± 0.027, respectively. The small spicule is 0.120 mm ± 0.012 long and the large spicule is more chitinized and striated, 0.418 mm ± 0.065 long. The gubernacle is 0.057 mm ± 0.009 long. The distance from the anus to the posterior end is 0.678 mm ± 0.089. The tail length is 0.678 mm ± 0.089. The cloacal opening is surrounded by seven pairs of papillae, of which four are pre-cloacal (three ventral and one lateral) and three post-cloacal (two ventral and one lateral). There are also five terminal papillae, four of which are arranged in two pairs and one close to the insertion of the tail.

Females (n=10): Total length 4.00 mm ± 0.253, body width at junction of esophagus to intestine 0.419 mm ± 0.052, length of anterior and posterior portions of esophagus are 0.189 mm ± 0.013 and 0.374 mm ± 0.031, respectively. The distance from the excretory pore and cervical papillae to the anterior end is 0.500 mm ± 0.053 and 0.186 mm ± 0.020, respectively. The distance from vulva to anus is 0.086 mm ± 0.016. Length from vulva to posterior end is 0.788 mm ± 0.082.

*Klossinemella* sp. 1 is similar to *K. conciliatus*, except for some morphometric values, the number and arrangement of caudal papillae, and the presence of wing structures. The nerve ring was not clearly observed but is likely to be located in the same region as that in *K. conciliatus*. *Klossinemella* sp. 1 was obtained from the stomach, small and large intestine of the big-headed Amazon River turtle.

### *Klossinemella* sp. 2 (Cosmocercoidea: Atractidae)

Cylindrical body with a transversely striated cuticle and large lateral wings beginning at the level of the posterior esophagus and continuing along the body until the insertion of the tail. Shape and disposition of the body resembled with that of a “seahorse” (Figure 6), with a loop in the posterior portion. Ornamented cuticle with papillae-like structures and a wider band at each striae portion Mouth with two bilobed lips, with each lobe having a small indentation forming smaller lobes. Highly modified Y-shaped interlips observed between the lips. Presence of nerve ring, cervical papillae, and excretory pore in the initial, middle, and final portions of the posterior esophagus, respectively. Presence of spinal structures in the posterior dorsal region of the body, extended till the beginning of the tail. Presence of a few spines in the ventral region after the anus. Viviparous females with a very pronounced vulva close to the anus. The tail was observed to be triangular, elongated, and filiform at its end.

Females (n=10): Total length 2.851 mm ± 0.341, body width at junction of esophagus to intestine 0.068 mm ± 0.004, length of anterior and posterior portions of esophagus are 0.142 mm ± 0.011 and 0.262 mm ± 0.024, respectively. The distance from the excretory pore to the anterior end is 0.331 mm ± 0.040. The distance from vulva to anus is 0.070 mm ± 0.004. Length from vulva to posterior end is 0.790 mm ± 0.097.

*Klossinemella* sp. 2 presented features characteristic of its genus; however, it was different from *K. conciliatus* and *Klossinemella* sp. 1 in having spines in the posterior dorsal region of the body; the other two species did not show this feature. *Klossinemella* sp. 2 was also obtained from the stomach, small and large intestine of the big-headed Amazon River turtle.

### *Klossinemella* sp. 3 (Cosmocercoidea: Atractidae)

**Figure 7:**
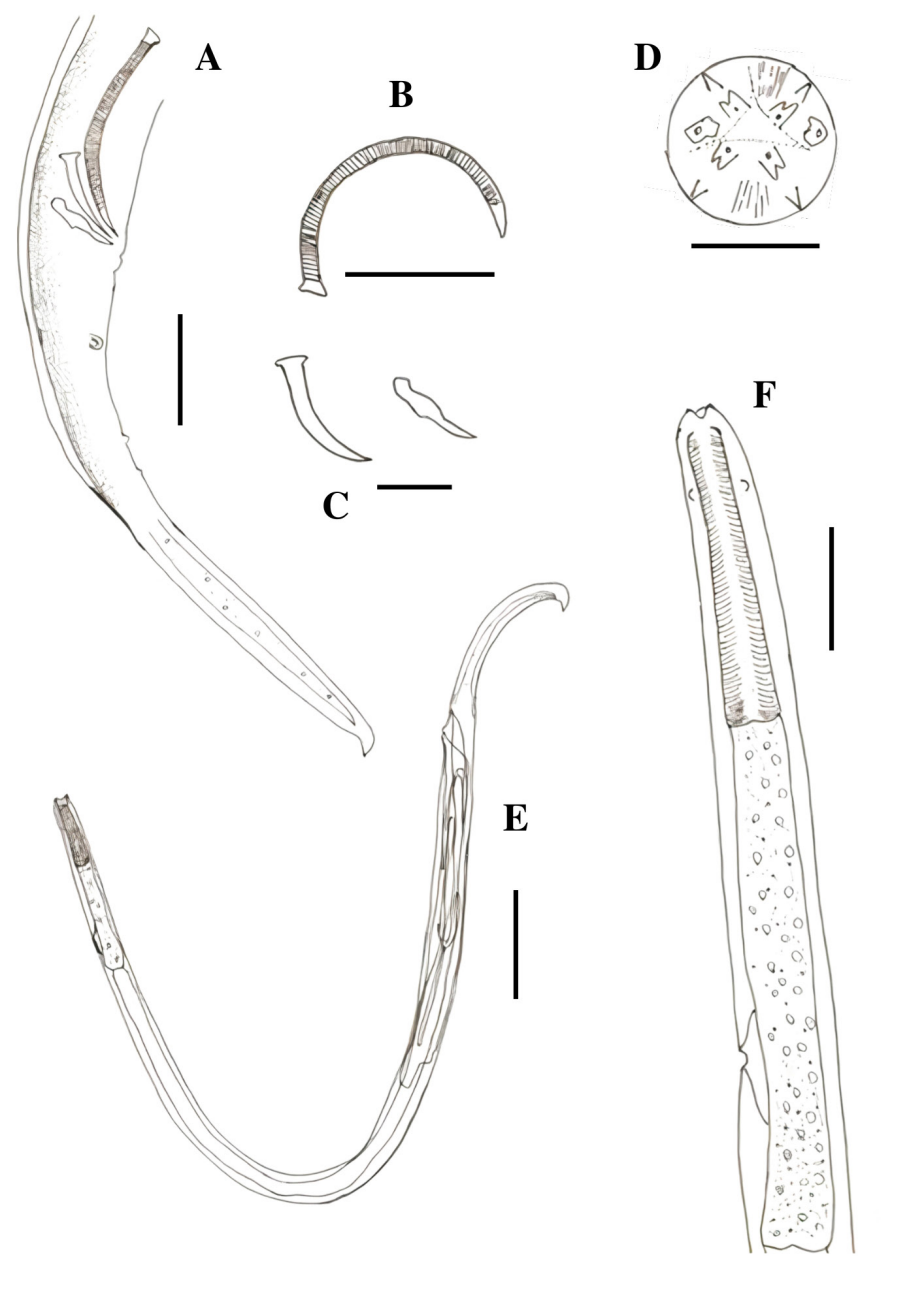
*Klossinemella* sp. 3. A. *Klossinemella* sp. 3, posterior end of the male with presence of three pairs of ventral postcloacal papillae. B. *Klossinemella* sp. 03, major spicule striated. C. *Klossinemella* sp. 3, smaller minor spicule and gubernacule. D. *Klossinemella* sp. 3, apical view of mouth with Y-shaped chitinized structures. E. *Klossinemella* sp. 3, female prodelphic specimen with larvae inside. F. *Klossinemella* sp. 3, detail of esophagus with bulbar region and excretory pore in the bulbar region, presence of cervical papillae and nerve ring. Scales: A, B, F: 0.05 mm; C: 0.025 mm; D: 0.018 mm.

Cylindrical body with a transversely striated fine cuticle from the beginning of the anterior end till the beginning of the muscular esophagus, and a smooth cuticle in the rest of the body. Absence of lateral wings. Males and females of similar sizes. Mouth with two bilobed lips, with each lobe having a small indentation forming smaller lobes. Highly modified Y-shaped interlips observed between the lips. Esophagus with two portions and a discrete bulb. Nerve ring and cervical papillae were difficult to observe. Excretory pore located in the final third of the glandular esophagus. Males with their posterior ends curled with two highly unequal spicules. Gubernacule present. Male and female tails were slightly triangular and elongated, with short, curved terminations. Viviparous females with their vulva close to the anus (Figure 7).

Males (n=10): Total length 2.174 mm ± 0.115, body width at junction of esophagus to intestine 0.046 mm ± 0.003, anterior esophagus length 0.134 mm ± 0.002, posterior esophagus length 0.203 mm ± 0.019. The distance from the excretory pore to the anterior end is 0.276 mm ± 0.029. The small spicule is 0.0.047 mm ± 0.002 long and the large spicule is more chitinized and striated, 0.116 mm ± 0.009 long. The gubernacule is 0.033 mm ± 0.004 long. The distance from the anus to the posterior end is 0.276 mm ± 0.027. It has three ventral post-cloacal pairs of papillae.

Females (n=10): Total length 2.323 mm ± 0.144, body width at junction of esophagus to intestine 0.044 mm ± 0.008, length of anterior and posterior portions of esophagus are 0.118 mm ± 0.008 and 0.217 mm ± 0.016, respectively. The distance from the excretory pore to the anterior end is 0.274 mm ± 0.030. The distance from vulva to anus is 0.049 mm ± 0.005. Length from vulva to posterior end is 0.403 mm ± 0.027.

*Klossinemella* sp. 3 share the same features of the genus *Klossinemella*, although it is markedly smaller than the other species of the genus, it does not have lateral wings and it was not possible to observe the nerve ring nor the cervical papillae to confirm their presence. Other differences are the number of caudal papillae and the subulate shape tail ending in a small, non-tapered curve. *Klossinemella* sp. 3 was obtained from the stomach, small and large intestine of the big-headed Amazon River turtle.

### *Nematophila grandis* (Diesing, 1839) Travassos, 1934 (Paramphistomoidea: Cladorchiidae)

Body flattened, large, and excavated ventrally. Acetabulum in the terminal position, oral sucker, presence of rudimentary diverticula in the oral sucker, muscular pharynx, and short esophagus. Slightly sinuous ceca reaching the acetabular region. Genital pore bifurcated and without suckers. Rudimentary cirrus sac and testicles. Dorsal uterus and lateral extracecal vitellaria arranged in small and numerous follicles (Figure 2). This parasite was recovered from the stomach, small and large intestine of *P. dumerilianus*.

*Nematophila grandis* has been described in several aquatic turtles in Central and South America (Dyer & Carr, 1990; Lombardero & Moriena, 1977; Sánchez et al., 2006; Salízar & Sanchez, 2004). In Brazil, it has been described in the Scorpion mud turtle (*Kinosternon scorpioides scorpioides*) (Alho, 1964) and Geoffroy’s toadhead turtle (*Phrynops geoffroanus*) (Fonseca et al., 2021). It has also been described in *P. dumerilianus* without any records of specific locations (Diesing, 1850). This digenean forms cercariae, called amphistomocercariae, which have pharynx, simple tail, and acetabulum in the posterior region of the body (Travassos et al., 1969). This is a new record location for this species.

### *Helicotrema spirale* Diesing, 1850 (Schistosomatoidea: Liolopidae)

Body flattened and elongated, with lateral ends curved toward the inside of the body, smooth cuticle, small oral sucker, testicules in the median region of the body, and intertesticular ovary. Short esophagus, pronounced pharynx, narrow intestinal ceca extending to the caudal end, and genital pore was pre-testicular. Large cirrus sac, and cecal and intercecal vitellaria (Figure 2). This parasite was recovered from the small intestine of *P. dumerilianus*.

*Helicotrema spirale* has also been described in the stomach and small intestine of the yellow-footed tortoise from Peru (*Chelonoidis denticulatus*) (Julca et al., 2014) and the small intestine of the yellow-spotted Amazon River turtle (*P. unifilis*) (Tantaleán & Forlong, 2013). In Brazil, it has been described in the common green iguana (*Iguana iguana*) and big-headed Amazon River turtle (*P. dumerilianus*), without any records of specific locations (Fernandes & Kohn, 2014). This is a new record location for this species.

### *Telorchis hagmanni* Lent & Freitas, 1937 (Plagiorchioidea: Telorchiidae)

General description: Body flattened, elongated, and covered by a cuticle with spines. The oral sucker was observed to be subterminal. The intestinal ceca were long and wide in the pre-acetabular region, ending close to the caudal end. Acetabular genital pore slightly displaced from the midline. A slightly elongated cirrus sac located in the acetabular and post-acetabular zones. Vitellaria formed from large follicules and located near the posterior border of the ovary. The testicules were located post-uterine in the posterior part of the body, and the uterus had many transverse loops.

*Telorchis hagmanni* has been described in the intestines of the yellow-spotted Amazon River turtle (*P. unifilis*), in the intestines of the giant South American turtle (*P. expansa*), and in the stomach of the Rio Magdalena River turtle (*Podocnemis lewyana*), with a very low prevalence in all cases (Brooks, 1976; Lenis & Vélez, 2011; Tantaleán et al., 2011). Likewise, we observed a prevalence of 4.76% (CI: 0.84-22.67%). This is the first record of *T. hagmanni* in *P. dumerilianus*, and a new record location for this species.

We found no statistical differences between the total parasite intensity and weight and length of the shell, or animal weight. Since Cosmocercoidea nematodes, such as oxyurids, may be commensals of this turtle species, it was expected that there would be little to no effect on their health, as has been observed in other reptiles (Telford, 1971; Hedley et al., 2013). Thus, when disregarding the species belonging to Cosmocercoidea, a medium-low interaction was observed between the parasite intensity and shell length (R = 0.4894, P = 0.0285). Similar results were obtained when only the digenean parasites were considered (R = 0.4972, P = 0.0257). As *Ancyrancanthus pinnatifidus* and digeneans have heteroxenous cycles and given that a successful infection is related to a susceptible host being exposed to an infective parasite, it can be assumed that turtles with a greater shell length have a higher parasite load as they are older and have had a longer exposure to the parasites. In fact, there is scarce information about *Ancyracanthus* spp. life cycle and some authors assume that some freshwater crustaceans serve as their obligate intermediate hosts (Moravec, 2007). Likewise, a wide variety of mollusks are the intermediate hosts of most digenetic parasites, after which, they undergo a short hostless free-living stage in which they percutaneously penetrate their definitive host, or a longer encysted stage in aquatic plants waiting to be ingested for their definite host (Cribb et al., 2014). *Peltocephalus dumerialianus* has an omnivore opportunistic behavior, being primarily herbivore (aquatic plants, algae, fruits, and seeds) and complementing its diet with a 5.24 to 51.7% of aquatic invertebrates and fish (Gentil et al., 2021; De La Ossa et al., 2011; Pérez-Emán & Paolillo, 1997).

Similarly, the correlation between the total parasite intensity and sex (male vs. female) was not significant. Nonetheless, when Cosmocercoidea data were removed, males were observed to have a higher mean parasite load (P = 0.0142, U = 19.00). The same was observed when only digenean parasites were considered (P = 0.0116, U = 18.00). The tendency of males to have a higher parasitic intensity may be related to sex-specific behavior, habitat use, diet, or the negative effects of male sex steroid hormones on the immune system (Hillgarth & Wingfield, 1997; Brown & Symondson, 2014). However, the ecoimmunology of reptiles is still being debated and analyzed to understand the link between an individual animal, its immune responses, and the environment (Field et al., 2022). Specific research in this regard is still necessary for the big-headed Amazon River Turtle, however it has been studied in other species.

For example, Saad & Shoukrey (1988) have observed that sex hormones could influence the immune system of hissing sand snakes, *Psammophis sibilans* (Linnaeus, 1758), mainly by stimulating it in females and suppressing it in males. In addition to immunosuppression, male androgens can directly affect parasite growth and development (Beckage, 1993; Brown & Symondson, 2014).

In *P. unifilis* was described a primarily herbivore diet in hatchling and adults, being that smaller turtles ate more animal matter than the larger size class (Teran et al., 1995). This diet behavior could be related to different exposition degrees during their lifetime. For *P. unifilis*, mature females are larger than males (Miorando et al., 2015), however, for *P. dumerilianus* adult males are slightly larger than females (Vogt & Iverson, 2002). Males had a carapace length of 46.1 ± 3.2 cm and females 37.1 ± 5.8 cm, there was more male adults (n=10) than female adults (n=4) and as we shown, carapace length could be related to a longer exposure to parasites (Table 2).

**Table 2:** Carapace length and weight by sex and age of *Peltocephalus dumerialianus* from the Jaú National Park, Barcelos, State of Amazonas, Brazil.

	Male (n=12)	Female (n=9)	Juvenile (n=7)	Adult (n=14)
Carapace length	46.1 ± 3.2	37.1 ± 5.8	35.1 ± 4.9	45.8 ± 3.1
Weight	8.7 ± 2.5	6 ± 2.7	4.9 ± 2.3	8.8 ± 1.9

Usually in parasitological studies, the extent of territorial displacement is considered as an underlying factor for the difference in parasite burden between male and female hosts (Ezenwa, 2004; Habig et al., 2018; Waterman et al., 2014). Nonetheless, it has been shown that the female *P. dumerilianus* displaces more than the male during the reproductive season, thereby increasing food diversity as well as the probability of being exposed to parasites (De La Ossa & Vogt, 2011; De La Ossa et al., 2011).

The higher parasite load in males, excluding the monoxenous Cosmocercoidea, can be explained by the larger contact area for parasites or larger amounts of ingested intermediate hosts (Poulin, 1996; Brown & Symondson, 2014), especially since crustaceans, mollusks and aquatic plants are a great part of *P. dumerilianus* diet and they are involved in *Ancyrancanthus pinnatifidus* and digeneans life cycles.

## Conclusions

This is the first record of *A. trematophila*, *K. conciliatus*, and *T. hagmanni* in *P. dumerilianus* and a new location record for *A. trematophila*, *P. hystrix*, *N. grandis*, *H. spirale*, and *T. hagmanni.* We observed a positive correlation between shell length and parasite burden of heteroxenic parasites, with males having a higher parasite load than females. We have also identified three possible new taxa, viz. *Klossinemella* sp. 01, *Klossinemella* sp. 02, and *Klossinemella* sp. 03.
